# Restricted Use of Echocardiography in Suspected Endocarditis during COVID-19 Lockdown: A Multidisciplinary Team Approach

**DOI:** 10.1155/2021/5565200

**Published:** 2021-07-30

**Authors:** Adam Hartley, Ahmed El-Sayed, Aula Abbara, Jamie Henderson, Anan Ghazy, Frances Davies, James R. Price, Prakash P. Punjabi, Jon Anderson, Roberto Casula, Hafiz Naderi, Perviz Asaria, Nilesh Sutaria, Iqbal S. Malik, Punam A. Pabari, Bushra S. Rana

**Affiliations:** ^1^National Heart and Lung Institute, Imperial College London, London, UK; ^2^Imperial College Healthcare NHS Trust, London, UK; ^3^NIHR Health Protection Research Unit in HCAI and AMR, Imperial College London, London, UK

## Abstract

**Background:**

Infective endocarditis (IE) is challenging to manage in the COVID-19 lockdown period, in part given its reliance on echocardiography for diagnosis and management and the associated virus transmission risks to patients and healthcare workers. This study assesses utilisation of the endocarditis team (ET) in limiting routine echocardiography, especially transoesophageal echocardiography (TOE), in patients with suspected IE, and explores the effect on clinical outcomes.

**Methods:**

All patients discussed at the ET meeting at Imperial College Healthcare NHS Trust during the first lockdown in the UK (23 March to 8 July 2020) were prospectively included and analysed in this observational study.

**Results:**

In total, 38 patients were referred for ET review (71% male, median age 54 [interquartile range 48, 65.5] years). At the time of ET discussion, 21% had no echo imaging, 16% had point-of-care ultrasound only, and 63% had formal TTE. In total, only 16% underwent TOE. The ability of echocardiography, in those where it was performed, to affect IE diagnosis according to the Modified Duke Criteria was significant (*p*=0.0099); however, sensitivity was not affected. All-cause mortality was 17% at 30 days and 25% at 12 months from ET discussion in those with confirmed IE.

**Conclusion:**

Limiting echocardiography in patients with a low pretest probability (not probable or definite IE according to the Modified Duke Criteria) did not affect the diagnostic ability of the Modified Duke Criteria to rule out IE in this small study. Moreover, restricting nonessential echocardiography, and importantly TOE, in patients with suspected IE through use of the ET did not impact all-cause mortality.

## 1. Introduction

Infective endocarditis (IE) is a severe multisystem disease, which has a high rate of morbidity and mortality [1]. This disease poses a significant challenge for effective healthcare delivery during pandemic conditions, owing to its insidious clinical presentation and reliance on imaging modalities [2]. Even noninvasive testing, such as transthoracic echocardiography (TTE), is problematic during the COVID-19 era, due to potential facilitation of virus transmission between patients and healthcare workers. Moreover, transoesophageal echocardiography (TOE) poses greater challenges as it is categorised as an aerosol-generating procedure, placing the operators at higher risk [3]. Yet, TOE is a class I indication for diagnosis in suspected IE but with a negative TTE, or for ruling out local complications with a positive TTE [2]. Although the diagnosis of IE is based upon a scoring system that includes clinical, serological, and imaging parameters, the Modified Duke Criteria [4], echocardiographic evidence of endocarditis is classed as a major diagnostic criterion.

There is, therefore, a paradox between guideline-driven optimal management and the ability of healthcare systems to deliver best practice during the COVID-19 pandemic. One approach is to limit investigations and only perform them when they directly influence patient management. The role of the “endocarditis team” (ET) has been shown to significantly reduce mortality and improve longer-term outcomes in IE [5]. We present our experience of utilising our established ET during COVID-19 lockdown to assess the appropriateness of cardiovascular imaging in patients with suspected IE, limiting imaging to cases where it is deemed essential.

## 2. Methods

### 2.1. Setting and Study Population

We conducted a prospective observational cohort study for all cases of suspected IE that were referred to the ET at Imperial College Healthcare NHS Trust (ICHNT) from the onset of lockdown in the UK (23 March 2020) until the 8 July 2020. The ICHNT is one of the largest hospital groups in the UK, which comprises three acute hospitals with 1,400 beds and receives referrals from several district general hospitals for specialist cardiology and cardiothoracic services. The ET consists of experienced consultants in infectious diseases, microbiology, cardiology, and cardiothoracic surgery. The ET reviews cases weekly, and a minimum of one consultant from each specialty is present, although frequently there are several from each specialty. Referrals for discussion at the meeting are received electronically from inpatient medical clinical teams. Approval for this project was granted by the Audit Department at Imperial College NHS Healthcare Trust (Reference No. CAR_029).

All cases aged 18 years and above were prospectively included in this observational study. Anonymised data were collected including patient demographics and individually examined for ET meeting outcome, imaging modalities performed, Modified Duke Criteria [4] with and without imaging parameters, and whether there were other clear clinical sources of possible infection, as well as 30-day and 12-month clinical outcomes (all-cause mortality) from the time of discussion.

### 2.2. Statistical Analysis

Continuous nonnormally distributed variables are presented as median with interquartile range (IQR). Categorical variables are presented as numbers with percentages. Chi-square or Fisher's exact test are used as appropriate to assess statistical significance with clinical parameters and IE diagnosis. A two-sided *p* value < 0.05 is considered statistically significant.

## 3. Results

During the first national lockdown in the UK, 38 patients were discussed at the ET meeting (Table 1). Of these, 27 (71%) were male with a median age of 54 (IQR 48–65.5) years. Eight (21%) had a prior history of IE, nine (24%) had previous prosthetic valve replacements, and three (8%) had a current/prior history of intravenous drug use. 11 (29%) had positive SARS-CoV-2 tests during the infective episode.

All of the patients were discussed during their inpatient hospital stay, except two who were outpatients with TTEs with concerning features for IE. The 36 patients cared for in hospital underwent workup consisting of routine blood tests, blood cultures, electrocardiograms, and chest x-rays.

At the time of ET discussion, eight (21%) had no echo imaging, six (16%) had point-of-care ultrasound (POCUS, performed on handheld devices with an ability for 2D and colour Doppler) only, and 24 (63%) had formal TTE. Throughout the IE episode in total, 33 (87%) had formal departmental TTE, six (16%) had TOE, two (5%) had cardiac MRI, and none had CT-PET. Five patients (13%) had no departmental echocardiographic imaging (Table 2). In those with departmental TTE, left ventricular systolic function was moderate or severely impaired in 6/33 (18%) and mildly impaired in 3/33 (9%) patients, whilst the remainder were reported to have preserved or good systolic function.

The ET meeting consensus decision was a diagnosis of IE in 12 (32%) and excluded IE in 26 (68%). Of the patients with confirmed IE, 11 (92%) were considered definite and one (8%) was possible IE according to the Modified Duke Criteria. Of these, 11 (92%) patients underwent formal TTE (one patient underwent POCUS only), whilst only four (33%) subsequently went on to have a TOE to assess the presence of local complications where surgery may be indicated. Specifically, TOE was performed in these patients to assess vegetation size and evidence of aortic root abscess, as well as to better assess valve morphology. Of the 26/38 patients where IE was ruled out by consensus ET decision, four (15%) did not have any imaging performed and two (8%) underwent TOE as a rule-out investigation. Of the 10 patients in whom IE was ruled out by the ET but with possible IE according to the Modified Duke Criteria, eight (80%) had departmental TTE whilst one (10%) went on to have a TOE following a TTE that was suggestive but inconclusive for IE. Two (20%) patients in this group had no imaging at all. Overall, six patients underwent TOE (16%), of which 1/6 (17%) tested positive for SARS-CoV-2.

Of the nine patients with prosthetic valves in the study, four had confirmed IE (44%). All patients with prosthetic valves underwent departmental TTE, and four (44%) underwent TOE. According to the Modified Duke Criteria, three were classified as definite IE, two were possible IE, and four excluded IE. 8/9 (89%) survived until discharge, whilst 6/9 (67%) were alive at one year. Repeat TTE imaging after discharge was undertaken in the referral centre for 5/8 (63%) of those alive at discharge, demonstrating no evidence of complications from possible IE. The median antibiotic duration in this cohort was 6 (IQR 2–9) weeks.

Of the patients with IE confirmed by the ET, 11/12 (92%) were related to either the mitral and/or aortic valves and 1/12 (8%) was related infection of the tricuspid valve. Four (33%) underwent surgical intervention. Surgery was performed in all of these patients owing to severe valvular regurgitation with either multiple large vegetations or evidence of embolic stroke.

Only 2/12 (17%) of the IE-confirmed patients had an alternative (i.e., non-endocarditis-related) possible source of infection, whilst 16/26 (62%) of the patients with IE ruled out by the ET had other potential infection sources. The relationship between an alternative source of infection and rejected IE was statistically significant (*p*=0.0354). Of these, possible infective sources were renal/urinary in 5/26 (19%) and skin in 4/26 (15%). Other rarer sources included indwelling lines, osteomyelitis, and necrotising pancreatitis.

**Figure 1:**
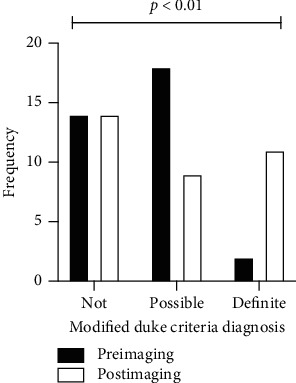
Change in the diagnostic category of the Modified Duke Criteria with and without echocardiography. The chi-square test used to assess significance, *p*=0.0099.

Of the 26 patients who did not have IE according to the ET, the Modified Duke Criteria for the 22 patients with some form of echocardiography performed was possible IE in 8 (36%) and not IE in 14 (64%). When comparing the Modified Duke Criteria with and without imaging parameters for these patients, utilising the standard cut offs, none were reclassified into different diagnostic groups. However, when analysing patients with confirmed IE, nine (75%) were reclassified from probable IE to definite IE. Therefore, the use of echocardiography did not alter the overall rule-out capability, or sensitivity, of the Modified Duke Criteria. However, echocardiography did increase the certainty of a positive diagnosis, and the overall ability of echocardiography to alter the Modified Duke Criteria score was statistically significant (*p*=0.0099, Figure 1).

At 30 days from the first ET discussion, all-cause mortality was 4/38 (11%) (2/12 (17%) of the patients with confirmed IE and 2/26 (8%) of the patients where IE was not diagnosed (aspiration pneumonia and bronchopneumonia)). Of the 10 patients with confirmed IE who survived until discharge, only two (20%) were readmitted to the referral hospital with an IE-related episode within the subsequent year. At 12-months from ET discussion, all-cause mortality was 11/38 (29%) (3/12 (25%) of those with confirmed IE and 8/26 (31%) of those where IE was not diagnosed). All of the patients undergoing surgical management for IE were alive at 12 months.

## 4. Discussion

Through utilisation of the ET at a large specialist centre, we were able to diagnose and treat patients with IE appropriately, without the need for routine traditional cardiovascular imaging. All-cause mortality at 30 days and 12 months from ET discussion is representative of larger cohorts during nonpandemic times (10–25% [6–9]). In addition, numbers of patients undergoing surgical management was not substantially lower than would be expected (33% versus 20–50% [6, 8]).

Echocardiography did not affect the ability to rule out an IE diagnosis in this study. This suggests that limiting echocardiographic investigation to those with possible/confirmed IE according to the Modified Duke Criteria (prior to the incorporation of the imaging parameters, but with standard diagnostic cutoffs) may be a safe and pragmatic approach to reduce nonessential imaging.

Importantly, reducing TOE, which is an aerosol-generating procedure and should be avoided in the majority of patients with COVID-19 [10] (only performed in six cases (16%)), did not confer any substantial increase in early mortality rate, albeit in this small study. Of note, the European Society of Cardiology guidance recommends all patients with suspected/proven endocarditis should undergo advanced imaging with TOE [2].

In this study, patients with a possible alternative source of infection were less likely to be diagnosed with IE. This underpins the central role the clinical assessment plays in this entity supported with routine basic investigations.

Previous studies have demonstrated the ability of echocardiography to confirm the presence of IE within the Modified Duke Criteria [11], but have not examined the rule-out ability within a group of patients with suspected IE. Clinical guidelines for cardiac imaging during the COVID-19 pandemic have reinforced the importance of echocardiography in the management of IE, especially with the postponement of routine dental visits, with an expected increase in incidence [12].

The main and most significant limitation of this study is its small size and single-centre nature. However, this was an unselected cohort, so it may be generalisable to a wider population under pandemic conditions. In addition, the study timeline was limited by the lockdown period, when access to echocardiography imaging was restricted.

## 5. Conclusions

In this study, we provide our real-world experience of the assessment of patients with suspected IE during the COVID-19 lockdown period. Cardiovascular imaging was limited via the ET, with the aim of reducing unnecessary patient contact and COVID-19 transmission, which did not significantly impact all-cause mortality at 30 days and 12 months. Importantly, significantly restricting TOE had no significant effect on mortality. In addition, not performing echocardiography in patients where the Modified Duke Criteria suggested IE was unlikely prior to imaging did not affect the overall ability of the Modified Duke Criteria to rule out IE in this study. Whilst our cohort is small, our data highlight the central role the ET assumes through bridging the gap when echocardiography is restricted.

## Figures and Tables

**Table 1:** Patient characteristics of the study population.

	*n* = 38
Age, years (median, interquartile range)	54 (48, 65.5)
Male, *n* (%)	27 (71%)
Ischaemic heart disease, *n* (%)	7 (18%)
Hypertension, *n* (%)	15 (39%)
Diabetes mellitus, *n* (%)	14 (37%)
Chronic lung disease, *n* (%)	3 (8%)
Chronic renal disease, *n* (%)	12 (32%)
Prior infective endocarditis, *n* (%)	8 (21%)
Prior prosthetic valve replacement, *n* (%)	9 (24%)
Current/prior history of intravenous drug use, *n* (%)	3 (8%)
Positive SARS-CoV-2 swab, *n* (%)	11 (29%)
Modified Duke Criteria (in whom echocardiography was performed)	*n* = 34
Major criteria	
Microbiology (blood cultures positive for IE)	20 (59%)
Imaging (echocardiogram positive for IE)	11 (32%)
Minor criteria	
Predisposition	17 (50%)
Fever	18 (53%)
Vascular phenomena (including those detected by imaging only)	11 (32%)
Immunological phenomena	1 (3%)
Microbiological evidence	3 (9%)

**Table 2:** Imaging modalities performed in the study population.

Imaging performed (%)	*n* = 38
Departmental transthoracic echocardiography	33 (87%)
Transoeseophageal echocardiography	6 (16%)
Cardiac magnetic resonance imaging	2 (5%)
Positron-emission tomography-computed tomography	0 (0%)
No departmental imaging performed	5 (13%)

## Data Availability

Data are available on appropriate request following review.
